# Supplementary material for: Conditional chemoconnectomics (cCCTomics) as a strategy for efficient and conditional targeting of chemical transmission
Source: eLife. 2024 Apr 30;12:RP91927. doi: 10.7554/eLife.91927 (PMC11060718; doi:10.7554/eLife.91927)
Supplement: Figure 3—source data 3. [file elife-91927-fig3-data3.zip › Figure 3 source data3 part 2.zip!/Figure 3-Source data 3-part2/1053-1-CRISPResso_Report_mpeqg9da.zip!/CRISPResso_on_1053_4/9.Alleles_frequency_table_around_sgRNA_ggagcggaatctacaaaacg.pdf]

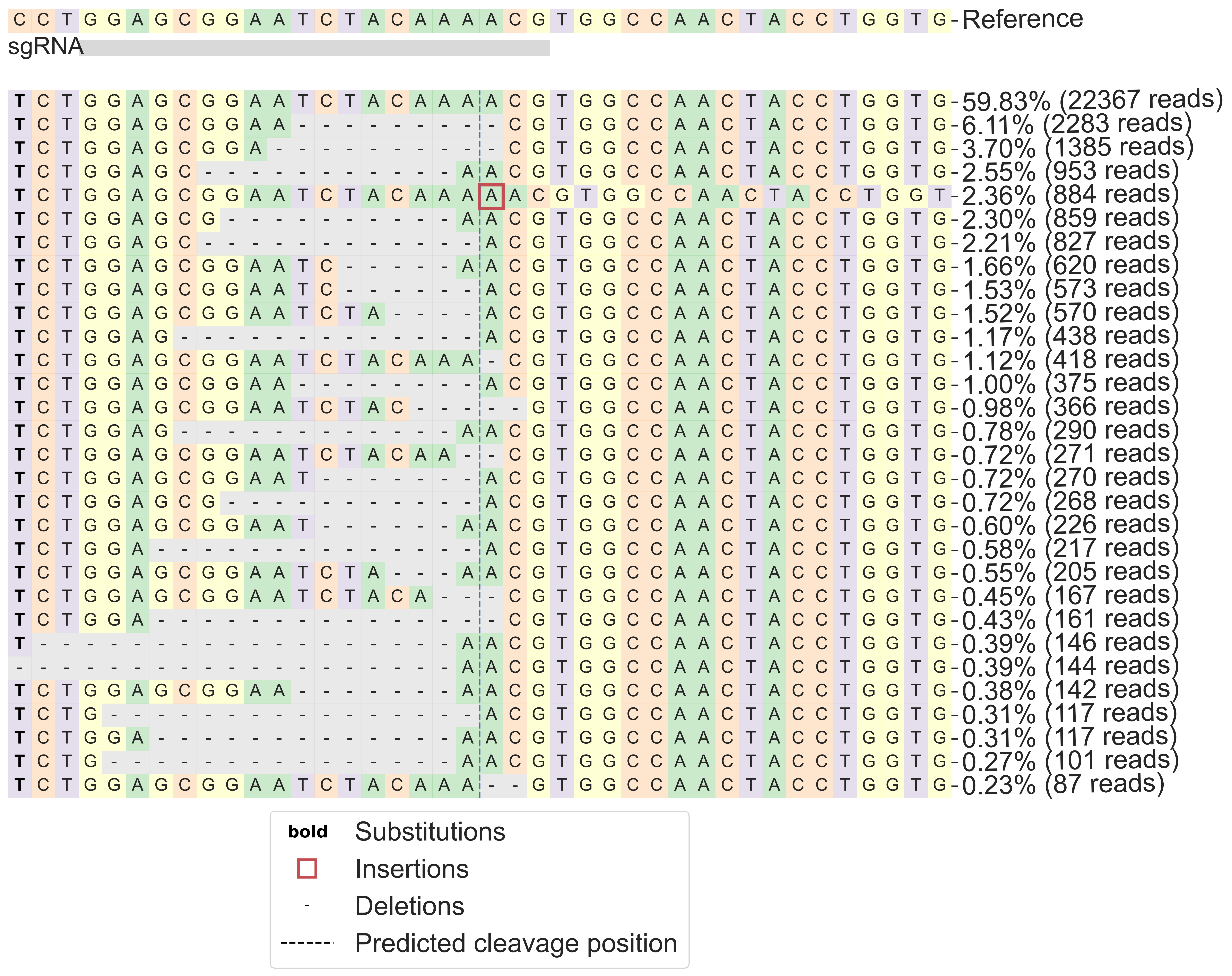

CCTGGAGCGGAATCTACAAAACGTGGCCAACTACCTGGTG Reference
sgRNA
59.83% (22367 reads)
6.11% (2283 reads)
3.70% (1385 reads)
2.55% (953 reads)
2.36% (884 reads)
2.30% (859 reads)
2.21% (827 reads)
1.66% (620 reads)
1.53% (573 reads)
1.52% (570 reads)
1.17% (438 reads)
1.12% (418 reads)
1.00% (375 reads)
0.98% (366 reads)
0.78% (290 reads)
0.72% (271 reads)
0.72% (270 reads)
0.72% (268 reads)
0.60% (226 reads)
0.58% (217 reads)
0.55% (205 reads)
0.45% (167 reads)
0.43% (161 reads)
0.39% (146 reads)
0.39% (144 reads)
0.38% (142 reads)
0.31% (117 reads)
0.31% (117 reads)
0.27% (101 reads)
0.23% (87 reads)
bold Substitutions
Insertions
Deletions
Predicted cleavage position
